# Treatment effects on adolescents at increased risk for psychosis: evaluation of a treatment approach combining a standardized manual with a smartphone app

**DOI:** 10.1192/j.eurpsy.2023.1317

**Published:** 2023-07-19

**Authors:** N. Traber-Walker, F. Probst, M. Gerstenberg, C. Bertossa, S. Walitza, M. Franscini

**Affiliations:** Department of Child and Adolescent Psychiatry and Psychotherapy, University of Zurich, Zurich, Switzerland

## Abstract

**Introduction:**

The goal of psychotic disorders has led researchers to focus on early identification of individuals at clinical high risk (CHR) for psychosis and treatment of CHR symptoms. CHR symptoms typically occur in adolescence and young adulthood. This is a very sensitive developmental period, and CHR-state is associated with increased functional impairment. Age-appropriate treatment approaches that address youth-specific interests, complex symptomatology, associated distress, and functional impairment are needed. However, there is a lack of research on treatment strategies for this vulnerable age group. To address this gap, we developed the combined treatment program “Robin” (standardized manual and smartphone app). The treatment program targets CHR symptoms, comorbid symptoms, and improvement of quality of life and daily functioning. The smartphone app “Robin Z” is an add-on treatment tool to support patients between their sessions. While a number of studies using smartphone apps in therapy have shown promising effects with adult psychosis patients, little is known about their use in therapy with minor patients. “Robin Z” is one of the first smartphone apps targeting adolescent patients with CHR or full-blown psychotic symptoms.

**Objectives:**

The investigation of efficacy of this specific intervention versus treatment as usual

**Methods:**

Our study was designed as a naturalistic clinical intervention study with a matched controlled design (treatment as usual). A total of 40 help-seeking adolescents (67% female) with CHR symptoms aged 13-18 years (mean age 15.86) were recruited to the intervention condition between September 2017 and May 2022. For the control group, data from 62 patients from a previous study are available and will be matched for age and gender. CHR symptoms, comorbid symptoms, functioning, self-efficacy, and quality of life will be monitored at six time points (baseline, during the treatment phase, immediately after the intervention, and 6, 12, and 24 months later).

**Results:**

All participants have now completed the intervention phase. In Paris, the first results on treatment effects will be presented at the symposium. This will include baseline data for the intervention group and their intraindividual changes in symptomatology, well-being, and level of functioning during and immediately after treatment. In addition, the results of the first follow up examinations compared to the control group will be presented.

**Conclusions:**

To our knowledge, this is the first controlled trial to evaluate the effectiveness of a specific treatment for adolescents with early psychosis combined with a smartphone app. The results of our evaluation are of clinical importance and should provide essential information for both the field of eMental Health and the topic of early intervention in psychosis.

**Disclosure of Interest:**

None Declared

